# Supplementary material for: Patterns of Chromosomal Instability and Clonal Heterogeneity in Luminal B Breast Cancer: A Pilot Study
Source: Int J Mol Sci. 2024 Apr 19;25(8):4478. doi: 10.3390/ijms25084478 (PMC11049937; doi:10.3390/ijms25084478)
Supplement: Supplementary file 1 [file ijms-25-04478-s001.zip › ijms-2868378-supplementary/Supplementary Tables S1 and S2.pdf]

SUPPLEMENTARY MATERIALS

Table S1. Level of Chromosomal instability (CIN), Clonal Heterogeneity (True Diversity Index) and Aneuploidy observed in luminal B BC patients.

RS	PAT	CHR	CIN<br>%	CH<br>TD index	ANE<br>%
ER+/PR+/HER2-	B1	CEP2	43	3.3	63
		CEP3	48	3.4	63
		CEP8	66	4.7	44
		CEP11	17	1.7	82
		CEP15	40	2.4	85
		CEP17	49	2.7	80
		**Total**	**44**	**3.0**	**70**
	B2	CEP2	58	4.3	41
		CEP3	47	3.8	49
		CEP8	57	3.7	46
		CEP11	32	2.2	78
		CEP15	20	1.8	80
		CEP17	37	3.0	41
		**Total**	**42**	**3.1**	**56**
	B3	CEP2	50	4.2	52
		CEP3	42	3.3	50
		CEP8	43	3.2	58
		CEP11	34	2.3	82
		CEP15	37	2.3	81
		CEP17	42	2.3	90
		**Total**	**41**	**2.9**	**69**
	B4	CEP2	51	4.0	43
		CEP3	60	4.0	55
		CEP8	48	2.3	92
		CEP11	21	1.9	86
		CEP15	53	2.7	85
		CEP17	63	5.1	43
		**Total**	**49**	**3.3**	**67**
ER+/PR-/HER2-	B6	CEP2	60,61	4.1	51
		CEP3	49	4.5	39
		CEP8	62	4.3	48
		CEP11	30,3	2.2	69
		CEP15	15	1.6	80
		CEP17	67	3.8	46
		**Total**	**47**	**3.4**	**56**
	B8	CEP2	51	4.0	43

		CEP3	59	4.7	37
		CEP8	42	3.1	60
		CEP11	19	1.7	89
		CEP15	45	3.6	53
		CEP17	44	2.9	66
		**Total**	**43**	**3.3**	**58**
	B10	CEP2	40	2.5	80
		CEP3	39	3.0	46
		CEP8	46	3.1	50
		CEP11	21	1.9	71
		CEP15	30	2.3	70
		CEP17	21	1.9	57
		**Total**	**33**	**2.5**	**62**
**ER+/PR+/HER2+**	**B5**	CEP2	44	3.0	73
		CEP3	54	4.4	54
		CEP8	33	2.6	70
		CEP11	13	1.6	85
		CEP15	12	1.4	100
		CEP17	50	2.7	82
		**Total**	**34**	**2.6**	**77**
	**B7**	CEP2	38	2.9	47
		CEP3	58	4.4	43
		CEP8	62	4.4	48
		CEP11	32	2.2	84
		CEP15	64	3.9	52
		CEP17	56	3.5	64
		**Total**	**52**	**3.5**	**56**
	**B9**	CEP2	42	2.9	69
		CEP3	38	3.0	47
		CEP8	38	2.4	79
		CEP11	12	1.6	58
		CEP15	22	1.8	86
		CEP17	61	3.8	52
		**Total**	**36**	**2.6**	**65**

Notes: According to the CIN level (% CIN), each patient was classified as having low CIN (CIN=0-25%), intermediate CIN (CIN=26%-50%), high CIN (CIN=51%-70%), or extreme CIN (CIN>70%). Stable or unstable aneuploidy was determined for each chromosome. A chromosome was considered to have stable aneuploidy if more than 20% (>20%) of the cells have identical probe signal patterns, while a chromosome, with fewer than 20% (<20%) of the cells with identical probe signal patterns was considered to have unstable aneuploidy. **Abbreviations:** RS, Receptor Status; PAT, Patient; CHR, Chromosome; CIN, Chromosomal Instability; ANE, Aneuploidy.

Table S2. CEP copy number variation (gains and losses) observed in luminal B BC patients.

RS	PAT	CHR	% CNV	TOTAL CNV
**ER+/PR+/HER2-**	**B1**	CEP2	1.77	**1.17**
		CEP3	1.53	
		CEP8	1.41	
		CEP11	0.2	
		CEP15	0.47	
		CEP17	1.65	
	**B2**	CEP2	1.47	**0.93**
		CEP3	1.54	
		CEP8	1.22	
		CEP11	0.39	
		CEP15	0.24	
		CEP17	0.69	
	**B3**	CEP2	2.01	**1.14**
		CEP3	1.58	
		CEP8	0.76	
		CEP11	0.43	
		CEP15	0.45	
		CEP17	1.6	
	**B4**	CEP2	1.5	**1.78**
		CEP3	2.46	
		CEP8	1.55	
		CEP11	0.25	
		CEP15	1.66	
		CEP17	3.27	
**ER+/PR-/HER2-**	**B6**	CEP2	1.54	**1.15**
		CEP3	2.08	
		CEP8	1.51	
		CEP11	0.39	
		CEP15	0.18	
		CEP17	1.21	
	**B8**	CEP2	1.75	**1.09**
		CEP3	1.61	
		CEP8	0.7	
		CEP11	0.21	
		CEP15	0.87	
		CEP17	1.38	
	**B10**	CEP2	1.33	**0.82**
		CEP3	1.37	

		CEP8	1.27	
		CEP11	0.27	
		CEP15	0.4	
		CEP17	0.3	
ER+/PR+/HER2+	**B5**	CEP2	1.49	**0.93**
		CEP3	1.93	
		CEP8	0.5	
		CEP11	0.15	
		CEP15	0.12	
		CEP17	1.41	
	**B7**	CEP2	1.1	**1.28**
		CEP3	1.56	
		CEP8	1.65	
		CEP11	0.38	
		CEP15	1.41	
		CEP17	1.57	
	**B9**	CEP2	1.33	**0.83**
		CEP3	1.23	
		CEP8	0.47	
		CEP11	0.17	
		CEP15	0.25	
		CEP17	1.55	

Notes: CEP2, CEP3, CEP8, CEP11, CEP15 and CEP17 copy number variations (gains and losses) were evaluated in all cases. According to the criteria for CEP copy number gain (mean CEP count ≥3) and loss (mean CEP count <1.6). **Abbreviations:** RS, Receptor Status; PAT, Patients; CHR, Chromosome; CNV, Copy number variation

SUPPLEMENTARY FIGURE S1 CAPTION

Figure S1. Multivariate analysis with Pearson correlation coefficient between clonal heterogeneity (CH) and clinicopathologic characteristics. Values greater than 0.5 are indicative of a statistically significant correlation. No correlation was found between CH with any of the variables studied: histotype (HT), tumor size (T), lymph nodes (N), lymphovascular invasion (LI), progesterone receptor (PR), HER2 and KI67.
