# Supplementary figures and images for: Patterns of Chromosomal Instability and Clonal Heterogeneity in Luminal B Breast Cancer: A Pilot Study
Source: Int J Mol Sci. 2024 Apr 19;25(8):4478. doi: 10.3390/ijms25084478 (PMC11049937; doi:10.3390/ijms25084478)

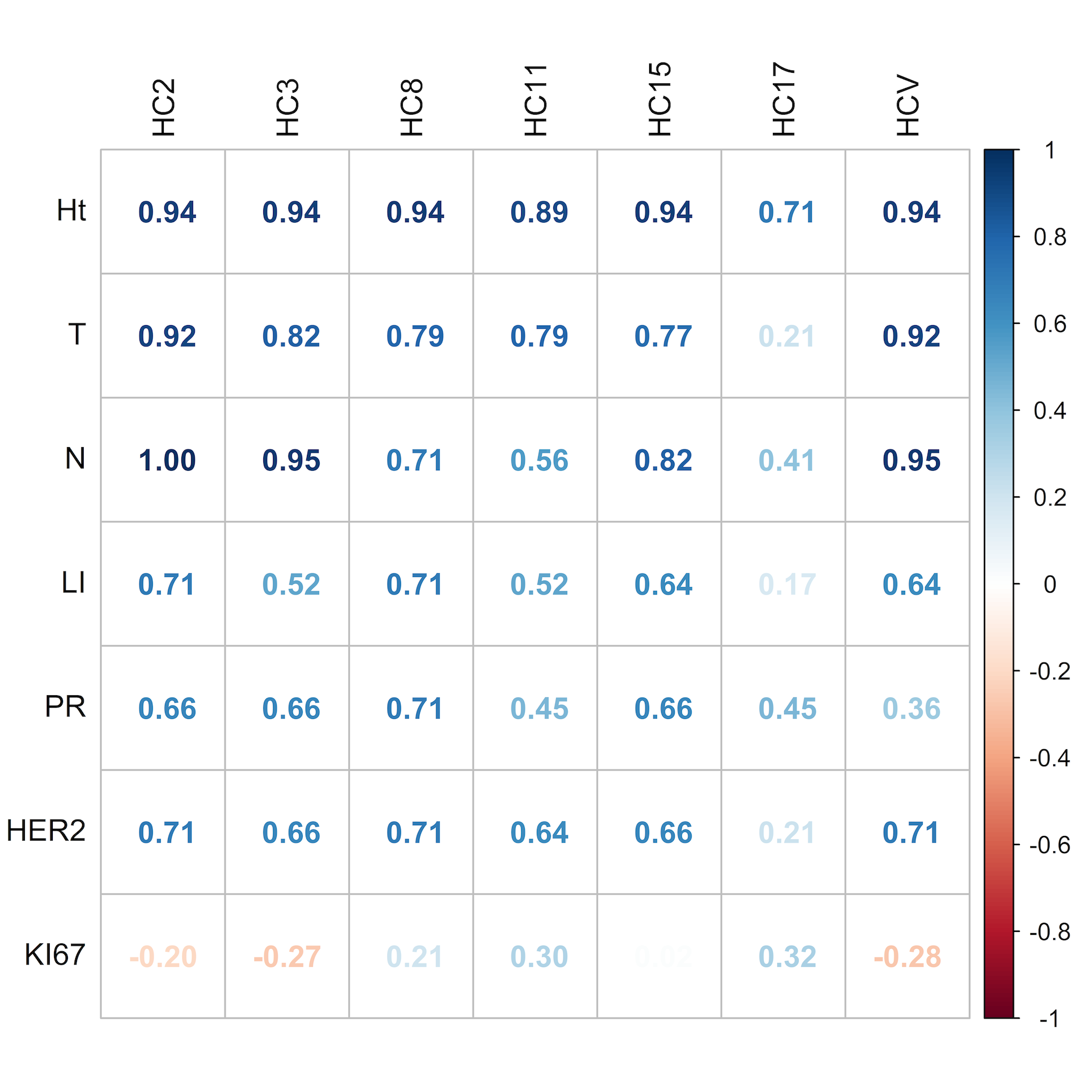

Supplement: Supplementary file 1 [file ijms-25-04478-s001.zip › ijms-2868378-supplementary/Supplementary Figure S1.tiff]
